# Heterozygous rare genetic variants in non-syndromic early-onset obesity

**DOI:** 10.1038/s41366-019-0357-5

**Published:** 2019-03-29

**Authors:** Clara Serra-Juhé, Gabriel Á. Martos-Moreno, Francesc Bou de Pieri, Raquel Flores, Julie A. Chowen, Luis A. Pérez-Jurado, Jesús Argente

**Affiliations:** 1 Genetics Unit, Universitat Pompeu Fabra, Hospital del Mar Research Institute (IMIM), C/Doctor Aiguader, 8, 08003 Barcelona, Spain; 2 Centro de Investigación Biomédica en Red de Enfermedades Raras (CIBERER), Instituto de Salud Carlos III, C/Sinesio Delgado, 4, 28029 Madrid, Spain; 3 Hospital Infantil Universitario Niño Jesús, Department of Endocrinology, Instituto de Investigación La Princesa, Universidad Autónoma de Madrid, Department of Pediatrics, Avenida Menéndez Pelayo, 65, 28009 Madrid, Spain; 4 CIBER de Fisiopatología de la Obesidad y Nutriciόn (CIBEROBN), Instituto de Salud Carlos III, C/Sinesio Delgado, 4, 28029 Madrid, Spain; 5 Women’s and Children’s Hospital, South Australia Medical and Health Research Institute (SAMHRI) and University of Adelaide, 72 King William Road, North Adelaide, SA 5006 Australia; 6 IMDEA Food Institute, CEIUAM + CSI, Crta. de Cantoblanco, 8, 28049 Madrid, Spain

**Keywords:** Endocrinology, Endocrine system and metabolic diseases

## Abstract

**Background:**

Obesity is a very heterogeneous disorder at both the clinical and molecular levels and with high heritability. Several monogenic forms and genes with strong effects have been identified for non-syndromic severe obesity. Novel therapeutic interventions are in development for some genetic forms, emphasizing the importance of determining genetic contributions.

**Objective:**

We aimed to define the contribution of rare single-nucleotide genetic variants (RSVs) in candidate genes to non-syndromic severe early-onset obesity (EOO; body mass index (BMI) >+3 standard deviation score, <3 years).

**Methods:**

Using a pooled DNA-sequencing approach, we screened for RSVs in 15 obesity candidate genes in a series of 463 EOO patients and 480 controls. We also analysed exome data from 293 EOO patients from the “Viva la Familia” (VLF) study as a replication dataset.

**Results:**

Likely or known pathogenic RSVs were identified in 23 patients (5.0%), with 7 of the 15 genes (*BDNF*, *FTO*, *MC3R*, *MC4R*, *NEGR1*, *PPARG* and *SIM1*) harbouring RSVs only in cases (3.67%) and none in controls. All were heterozygous changes, either de novo (one in *BDNF*) or inherited from obese parents (seven maternal, three paternal), and no individual carried more than one variant. Results were replicated in the VLF study, where 4.10% of probands carried RSVs in the overrepresented genes. RSVs in five genes were either absent (*LEP*) or more common in controls than in cases (*ADRB3*, *LEPR*, *PCSK1* and *PCSK2*) in both obese datasets.

**Conclusions:**

Heterozygous RSVs in several candidate genes of the melanocortin pathway are found in ~5.0% patients with EOO. These results support the clinical utility of genetic testing to identify patients who might benefit from targeted therapeutic intervention.

## Introduction

Obesity is the most prevalent chronic childhood disease in the occidental world and a risk factor for later obesity- and metabolic-related disorders. It is a heterogeneous multifactorial disease with high heritability (50–75%) that is undoubtedly higher in severe early-onset cases [1–3]. The genetic causes underlying obesity remain largely unknown with an important proportion of information missing regarding its heritability.

A small percentage of obesity occurs as part of a syndromic entity [4–10], but in the majority, obesity is not accompanied by other specific phenotypes. Highly penetrant rare genetic variants, mainly autosomal recessive, affecting at least eight genes [*LEP* (MIM 164160), *LEPR* (MIM 601007), *MC4R* (MIM 155541), *PCSK1* (MIM 162150), *POMC* (MIM 176830), *MC3R* (MIM 155540), *SIM1* (MIM 603128) or *NTRK2* (MIM600456)] are reportedly found in 2–5% of non-syndromic obese patients [11–17]. However, multigenic or multifactorial inheritance is the best model to explain the aetiology of most cases. Common genetic variants involved in these forms of obesity have been identified by genome-wide and candidate-gene association studies. Genetic variants in genes such as *ADRB3* (MIM 109691), *BDNF* (MIM 113505), *FTO* (MIM 610966), *GHSR*, *NEGR1* (MIM 613173), *PCSK2* (MIM 162151), *PPARG* (MIM 601487) and *TMEM18* (MIM 613220) [18–22], among others, have been related to body mass index (BMI), but the strongest effect of a single common variant might explain about only 2% of the variance in BMI. Similar to other complex traits [23, 24], copy number variants (CNVs) also contribute to the genetic basis of obesity [25–28]. Identification of a potential genetic cause of severe obesity is important not only for individualized follow-up and genetic counselling but also because novel therapeutic approaches for genetically defined obesities are becoming available [29].

Next-generation sequencing (NGS) has facilitated identification of the molecular basis of Mendelian diseases, even with a reduced number of samples [30, 31]. However, large numbers of patients are needed to identify a significant mutation burden in a specific gene and prove its pathophysiological relevance in multifactorial or highly heterogeneous genetic disorders. Given the known population stratification, appropriate controls from the same geographic origin are also needed to discard population variants that might not be disease causative. Strategies using pooled DNA-sequencing have proven effective in minimizing the costs of tackling multifactorial or heterogeneous disorders using large numbers of samples [32].

In highly heterogeneous diseases such as obesity, severe cases with early onset are more likely explained by highly penetrant rare genetic variants. We have used a pooled strategy to sequence a panel of selected candidate genes in 463 patients with non-syndromic severe early-onset obesity (EOO) and 480 controls searching for rare sequence variants (RSVs) with high penetrance.

## Subjects and methods

### Subjects and samples

Severe early-onset obese patients were selected by using the criteria of a BMI *Z*-score above three according to age and sex for the Spanish population [33] at their first examination and that visited the clinic due to obesity that was reported to have an onset before 3 years of age.

Patients underwent a detailed clinical evaluation, including oriented clinical history, physical examination and complementary tests to rule out syndromic, endocrine or secondary forms of obesity. These evaluations included an interview with a dietitian to determine eating habits such as the frequency, speed and amount of food ingested. A molecular karyotype by microarray and a methylation-sensitive multiple-ligation probe amplification to discard specific entities that might clinically overlap with isolated obesity were also performed [5].

A sample of 463 subjects with severe EOO was selected. Blood was collected from patients and both parents when possible to study the segregation of the genetic variants with the phenotype in the family. DNA was isolated from total blood using the Gentra Puregene Blood kit (Qiagen) according to the manufacturer’s instructions.

A total of 480 adult subjects with normal weight (BMI <P75) from the same geographic origin, provided by Banco Nacional de ADN Carlos III (Universidad de Salamanca, Spain), were used as controls.

### Candidate genes

To define the list of 15 candidate genes, a systematic literature review was done to identify: (1) genes with reported mutations in patients with obesity (*LEP*, *LEPR*, *MC3R*, *MC4R*, *PCSK1, NTRK2* and *SIM1)* [11–16, 34], and (2) genes with single-nucleotide polymorphisms associated to obesity in genome-wide association studies (*BDNF*, *FTO*, *NEGR1*, *GHSR*, *ADRB3*, *PPARG*, *PCSK2* and *TMEM18*A) [18–22].

### Pooled DNA-sequencing

**Fig. 1:**
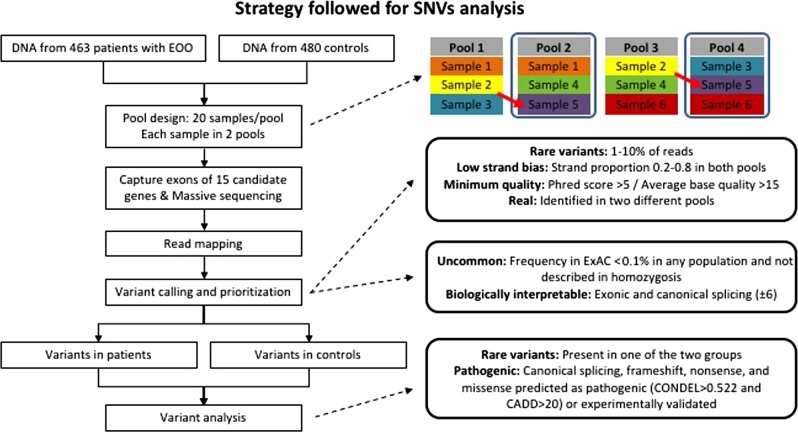
Summary of the strategy followed for single-nucleotide variant (SNV) analysis and rare sequence variant (RSV) identification

Targeted enrichment was used to capture coding regions from the 15 selected genes (SeqCap EZ Choice Enrichment Kits, Roche Sequencing). Instead of sequencing individual samples, a pooled DNA strategy was used. Each sample was included in two different pools and each pool had 20 different samples. A total of 48 pools from patients and 48 pools from controls were designed (Fig. 1). To identify which sample carried each variant, the results from each pool were overlapped to identify the two pools harbouring the same alterations. Using this pooling approach, one heterozygous variant in a sample is expected to appear in about 1/40 (2.5%) of the reads. To ensure that variants with a low percentage of reads were represented in the sequencing experiment, high coverage was mandatory. The massive sequencing was done with MiSeq (Illumina).

### Variant calling

Reads were mapped to the human reference genome (UCSC hg19). We used the MUTEC software [35] to call RSVs represented in a low percentage of reads. Given the very high coverage, false-positive calls were expected in almost all positions mapped. Thus, several quality filters were used to discriminate real variants from false positives in an automatic manner (Fig. 1) and visual inspection of reads in the integrated genome viewer [36] allowed us to clearly discriminate in dubious cases. Six samples with seven previously detected variants were used for optimization of filtering parameters. Base quality index and strand bias were the best predictors to reduce false-positive detection rates. Since false positives tended to have much lower base quality values, only variants with individual Phred scores over 5 and an average base quality over 15 in the two different pools were selected. Variants with high strand bias were found to be false positives. Thus, variants with extreme strand bias (proportion of same strand reads <20% or >80%) were discarded as likely false positives.

Custom-made Perl scripts were used to automate sample identification crossing the pools where the same variant was detected. The proportion of variants in each pool was also considered in the pipeline as an estimation of allele frequency. Although obesity is a highly heterogeneous disorder, the same RSV might appear twice (present in two alleles from either the same or different samples), expected in 5% of the reads. Consequently, the first step was filtering the variants present in between 1 and 10% of the mapped reads. Variants with a lower percentage were probably artefacts and variants with a higher percentage (present in three or more samples in the same pool) were thought too common to be considered highly penetrant RSVs for this heterogeneous disease.

In positions where the coverage was under 1000×, it was particularly difficult to discriminate between real variants and false positives. Therefore, we individually assessed all variants in positions with coverage under 1000×.

### Prioritization and analysis of variants

To establish the burden of RSVs identical approaches were used in each group (Fig. 1). Intronic variants were excluded due to the difficulty in interpreting their clinical significance and only exonic variants and those putatively affecting splicing (±6) were selected. We discarded variants with population frequencies in any public database (gnomAD, 1000 genomes, Kaviar, Spanish variant server) [37] higher than 1/1000 or described in a homozygous state in at least one individual, assuming that variants with a relatively high frequency in the general population are unlikely high penetrant variants related to the severe phenotype of EOO.

All nonsense or frameshift, canonical splicing and missense variants predicted as damaging (Condel >0.522 and CADD >20) [38, 39] were considered as highly likely to be pathogenic (from now on called likely pathogenic variants). All previously reported mutations with experimentally validated functional consequences were also included [40]. To define pathogenicity, we also considered the constraint metrics for variation tolerance of each gene. Variation-intolerant genes were considered those with pLi (probability of loss-of-function intolerance) >0.50 and missense *Z*-score >0 [37].

### Variant validation and co-segregation

To validate RSVs detected by pooled DNA-sequencing, PCR amplicons from individual DNA samples were obtained and sequenced by Sanger technology. The same technique was used to analyse parental samples when available to define inheritance pattern and possible co-segregation of the RSV with the phenotype in each family.

### Replication cohort

Exomes from 293 unrelated patients with EOO from the Viva la Familia (VLF) study [41] were used to replicate the results found in our initial cohort. VLF comprises obese probands with BMI >95th percentile, between 4 and 19 years old, from Hispanic families in Houston. Sequence read archive (SRA) files were downloaded from publicly available servers (dbGAP: phs000616.v2.p2). Variant calling in the 15 candidate genes was done with GATK [42] and filtering was performed using the same criteria as above.

### Ethics statement

The project was approved by the Medical Ethical Committee of Hospital Infantil Universitario Niño Jesús in Madrid, Spain and is in accordance with the “Ethical Principles for Medical Research Involving Human Subjects” adopted in the Declaration of Helsinki by the World Medical Association (64th WMA General Assembly, Fortaleza, Brazil, October 2013) and Spanish data protection act (Ley Orgánica 15/1999 de Protección de Datos). Written informed consent was obtained from all patients or their legal guardian after a complete description of the study.

## Results

### RSV detection sensitivity and specificity

The mean sequence coverage of patient pools was 2396× (SD: 574) and 3428× (SD: 660) in controls, with >98% of targeted sites showing coverage above 400×. The sensitivity and specificity of this approach were assessed. Six of the seven known variants in samples included in the study were blindly identified with the filters mentioned above, yielding a sensitivity of 86%. The only variant not identified had low-quality scores. As proof of concept for high specificity, 23 variants detected with the pooled DNA approach were selected and all (23/23, 100%) validated by Sanger sequencing. Although the pooled DNA approach may not detect a small percentage of real variants, it does not capture false positives. These results demonstrate that the strategy used is efficient to identify RSVs in large cohorts of samples.

### Burden of RSVs in EOO

A total of 73 different RSVs were selectively identified in only one group (Table 1): 43 variants in 48 subjects of the patient group and 30 variants in 31 controls. This difference was statistically significant (odds ratio (OR) = 1.61; *p* = 0.0342; Table 1 and Supplemental Tables 1 and 2). We used very stringent criteria of pathogenicity to better define the putative clinically relevant variants: all loss-of-function (nonsense, frameshift and canonical splice-site variants) and missense variants predicted as pathogenic by two stringent algorithms (Condel and CADD) were considered as likely pathogenic. Likely or known pathogenic (previously validated) RSVs were found in 23 patients and 8 controls (OR = 2.98; *p* = 0.0055). Most RSVs were present in a single sample, but a few cases with two samples harbouring the same RSV were found and three patients shared a variant in *FTO*.

**Table 1:** RSVs identified per gene in each one of the analysed groups: EOO-Sp, *n* = 463; VLF, *n* = 293; and controls, *n* = 480

	All RSVs			Likely pathogenic RSVs		
	EOO-Sp (%)	VLF (%)	Controls (%)	EOO-Sp (%)	VLF (%)	Controls (%)
*ADRB3*	1 (0.22)	1 (0.34)	4 (0.83)	–	–	1 (0.21)
***BDNF***	4 (0.86)	1 (0.34)	–	3 (0.65)	1 (0.34)	–
***FTO***	4 (0.86)	4 (1.37)	1 (0.21)	1 (0.22)	–	–
*GHSR*	2 (0.43)	6 (2.05)	3 (0.63)	–	1 (0.34)	–
*LEP*	–	–	–	–	–	–
*LEPR*	7 (1.51)	5 (1.71)	5 (1.04)	2 (0.43)	2 (0.68)	3 (0.63)
***MC3R***	3 (0.65)	3 (1.02)	–	2 (0.43)	1 (0.34)	–
***MC4R***	7 (1.51)	6 (2.05)	2 (0.42)	6 (1.30)	4 (1.37)	–
***NEGR1***	3 (0.65)	–	–	1 (0.22)	–	–
*NTRK2*	2 (0.43)	3 (1.02)	4 (0.83)	1 (0.22)	2 (0.68)	1 (0.21)
*PCSK1*	4 (0.86)	–	5 (1.04)	3 (0.65)	–	1 (0.21)
*PCSK2*	2 (0.43)	–	5 (1.04)	–	–	2 (0.42)
***PPARG***	3 (0.65)	–	–	2 (0.43)	–	–
***SIM1***	6 (1.30)	2 (0.68)	1 (0.21)	2 (0.43)	–	–
*TMEM18*	–	1 (0.34)	1 (0.21)	–	1 (0.34)	–
	**48 (10.37)**	**32 (10.92)**	**31 (6.46)**	**23 (4.97)**	**12 (4.10)**	**8 (1.67)**
**Selected genes**	30 (6.48)	16 (5.46)	4 (0.83)	17 (3.67)	6 (2.05)	–
Other genes	18 (3.89)	16 (5.46)	27 (5.63)	6 (1.30)	6 (2.05)	8 (1.67)

Bold represents the selected genes.

The number and percentage of individuals carrying rare and probably pathogenic genetic variants per gene are shown. The total burden of RSVs is significantly higher in EOO-Sp (48/463) than controls (31/480) (OR = 1.61; *p* = 0.0342), and in VLF compared to controls (32/293; OR = 1.69; *p* = 0.0306). The proportion of individuals carrying likely pathogenic RSVs was also significantly higher in EOO-Sp than in controls (23/463 vs. 8/480; OR = 2.98; *p* = 0.0055). Genes accumulating the RSV load in patients compared with controls are in bold: *BDNF*, *FTO*, *MC3R*, *MC4R*, *NEGR1*, *PPARG* and *SIM1*. The proportion of individuals carrying RSVs was significantly higher in EOO-Sp than in controls (30/463 vs. 4/480; OR = 7.78; *p* < 0.0001). This difference was also statistically significant in VLF (16/293; OR = 6.55; *p* = 0.0002). Considering only probably pathogenic variants, the proportion in patients (17/463 in EOO-Sp and 6/293 in VLF) was significantly higher than in controls (*p* < 0.0001 and *p* = 0.0029, respectively), where none were found (0/480).

*OR* odds ratio, *RSV* rare sequence variant, *OR* odds ratio, *EOO-Sp* early-onset obesity-Spain, *VLF* Viva la Familia

A subset of sequenced genes accumulated the differential mutational load found in patients compared to controls: *BDNF*, *FTO*, *MC3R*, *MC4R*, *NEGR1*, *PPARG* and *SIM1*. Analysing this group of genes separately, a total of 30 patients with RSVs were identified in patients versus four controls (OR = 7.78; *p* < 0.0001; Table 1) and four of these seven overrepresented genes (*BDNF*, *NEGR1*, *PPARG* and *SIM1*) are variation intolerant (Supplemental Table 3). Analysing the likely pathogenic variants in the subset of genes, RSVs were identified in 17 patients (3.67%) and no controls (*p* < 0.0001; Table 1). Two of the variants identified were present in two unrelated patients (Table 2). All 17 likely pathogenic variants identified in the overrepresented subset of genes heterozygous and no individual carried more than one likely pathogenic variant (Tables 1 and 2).

**Table 2:** Main clinical features of the patients harbouring likely pathogenic RSVs in the overrepresented subset of genes

Gender	Gene	Type	gDNA	cDNA	Protein	Inheritance	Age at exam (years)	BMI (SDS)	Height (SDS)	Target height (SDS)	Bone age acceleration (months)	Newborn weight (SDS)	Cognition and behaviour	Hyperphagia	Metabolic disturbances	Other comorbidities	Familial obesity background
Male	*MC4R*	Missense*	Chr18:58038768	815C>T	P272L*	Maternal	6.84	+6.5	+4.3	+0.10	26	+3.2	NR	+	IR	Precocious pubarche	Mother (infancy) not adult
Male		Missense*	Chr18:58038807	776C>T	A259V*	Maternal	6.92	+7.0	+1.2	−1.52	−11	+2.0	NR	+	None	Macroorchydism	Both parents and families
Male		Missense*	Chr18:58039134	449C>T	T150I*	–	9.58	+5.8	+1.8	−0.90	42	−1.0	NR	+	IR + IGT + HU	Liver steatosis	Both parents and families
Male		Missense*	Chr18:58039203	380C>T	S127L*	–	14.25	+14.0	−1.3	−2.33	14	−0.4	NR	+++	IR + HT + HU	None	Both parents and families
Male		Missense*	Chr18:58039356	227A>G	H76R*	Maternal	14.00	+5.8	+1.1	−0.81	33	+0.1	NR	+++	IR + HU	Liver steatosis	Both parents and paternal family (grandfather and 3 aunts)
Male		Nonsense	Chr18:58039519	64A>T	R22X	Maternal	5.00	+18.8	+1.0	−2.06	51	+5.7	NR	+++	IR + HT + HU	Liver steatosis	Both parents and maternal family (morbid)
Female	*MC3R*	Missense	Chr20:54824803	904C>T	R302W	–	11.16	+4.4	+0.8	+0.57	10	−0.2	NR	+	IR + HU	None	Both parents and families
Male		Missense	Chr20:54824803	904C>T	R302W	–	12.00	+3.8	−0.9	−1.00	4	−0.4	NR	+	None	None	Both parents and families
Female	*SIM1*	Missense	Chr6:100896482	616C>A	Q206K	Maternal	8.75	+5.6	+1.8	+0.48	12	−1.1	NR	+	IR + IGT	None	Father and sister
Male		Splicing	Chr6:100898138	352+1G>A	–	Paternal	8.66	+4.3	+0.7	−0.01	12	+1.16	NR	+	IR + IGT	None	Father and paternal family (grandmother, 2 uncles and 1 aunt)
Male	*PPARG*	Missense	Chr3:12422920	326T>A	I109N	Maternal	9.33	+5.5	+4.2	+1.06	24	+5.1	NR	+	IR	None	Father
Male		Missense	Chr3:12434173	457C>T	R153W	–	11.00	+4.1	−0.4	−1.57	0	−2.0	NR	+	IR + IGT + HT	None	Father and sister
Male	*BDNF*	Missense	Chr11:27679421	691A>G	I231V	Paternal	6.00	+3.9	+0.3	−0.76	-6	−0.4	NR	+	None	None	Mother and maternal family (grandfather)
Female		Missense	Chr11:27679421	691A>G	I231V	–	11.75	+3.3	−0.1	−1.18	3	+0.1	NR	+++	IR + HU	Liver steatosis	Mother
Female		Missense	Chr11:27679691	421T>G	C141G	De novo	11.16	+6.1	+2.0	−0.41	17	+0.1	NR	+	IR + HU	Liver steatosis	Brother and paternal family (aunt, grandfather)
Male	*NEGR1*	Missense	Chr1:72400858	313 A > G	I105V	Maternal	2.92	+8.8	+1.7	−0.11	3	+0.8	SP+BP	+++	HU	None	Both grandmothers
Female	FTO	Missense	Chr16:53859890	238 C > T	R80W	Paternal	6.66	+3.8	+1.1	−0.57	14	−1.7	NR	+++	IR + HT + HU	Liver steatosis	Sister, both parents and maternal family (grandmother, 2nd degree aunt)

In columns “Type” and “Protein” the * indicates functional information available

*SDS* standard deviation score, *BMI* body mass index, *gDNA* genomic DNA, *cDNA* complementary DNA, *NR* nothing relevant, *SP* speech delay, *BP* behavioural disturbances, *IR* insulin resistance, *IGT* impaired glucose tolerance, *HU* hyperuricaemia, *HT* hypertriglyceridaemia

In the remaining subset of genes, 18 RSVs were identified in patients and 27 in controls (not statistically significant), including 6 likely pathogenic variants in patients and 8 in controls (not statistically significant; Supplemental Tables 1 and 2).

### Heterozygous RSVs in patients with EOO

Among the genes with higher mutational load in patients, *MC4R* was the most frequent with seven RSVs in patients (1.51%). Of these, five have been reported as pathogenic by demonstration of partial (H76R) or complete (S127L, T150I, A259V and P272L) loss of function [13, 40]. The two RSVs not previously described include a nonsense change interrupting the protein in the 22nd codon (R22X) and a missense change (V52A) predicted as likely benign. In the five cases with parental samples available, the RSV was inherited from obese mothers. Two missense variants in *MC4R* were detected in the control group, both predicted as benign and not previously described in patients. Three patients were also found to have RSVs in a related gene, *MC3R*, with the likely pathogenic RSV (R302W) identified in two unrelated patients (Table 2).

*SIM1* also showed a high mutational load in patients (*n* = 6) compared to controls (*n* = 1). Two of the six variants identified in patients are strongly predicted to be pathogenic. One affects a canonical splice site in intron 3 (c.352 + 1G>A) and the other is a missense variant on a highly conserved residue of the first Perl–Arnt–Sim of the protein (Q206K) found in two siblings concordant with the phenotype. These RSVs were inherited from an obese father (splicing variant) and obese mother (missense variant) (Table 2). The variant detected in a control subject and four of the RSVs in patients are not predicted to be pathogenic by our criteria.

In the *BDNF* gene, three RSVs were found in four patients and none in controls. Two of the variants are predicted to be pathogenic. One of these RSVs was not found in parental samples, indicating that it occurred de novo in the patient. Parenthood was proven using microsatellite markers. The other RSV (I231V) was found in two unrelated cases, paternally inherited in the case with parental samples available (Table 2).

We detected three RSVs (two likely pathogenic) in *PPARG* in patients, as well as one single likely pathogenic missense RSV in *FTO* and *NEGR1*, and none in controls.

### Clinical features of RSV carriers

The main phenotypic features of patients harbouring likely pathogenic RSVs in the selected genes are detailed in Table 2. The six patients harbouring pathogenic RSVs in *MC4R* were males with a BMI above + 5.5 standard deviation score (SDS), orexigenic impulsivity, hyperinsulinism, some overgrowth (height >+1 SDS over target height) and advanced bone age (14 months on average), a phenotype previously defined in patients with *MC4R* mutations [43, 44]. The patient with the A259V variant had macroorchydism (8 cc volume testes despite Tanner stage I), which is not a common feature of MC4R deficiency, given the lack of *MC4R* expression in the testis [45]. The patient with the novel nonsense mutation (R22X) had an extremely high BMI (+18.8 SDS), uncontrollable orexigenic impulsivity, overgrowth (+3 SDS), advanced bone age (51 months over chronological age) and hyperinsulinism. His mother, also a carrier of the R22X mutation, had a BMI of 51.3 kg/m^2^. The patient with a predicted benign RSV at *MC4R* (V52A) was a girl with milder obesity (+4.2 BMI-SDS), no overgrowth (height at −1 SDS), average bone age, and hyperinsulinism.

The two patients with likely pathogenic RSVs in *MC3R* had milder obesity (BMI around +4 SDS) and no overgrowth. One had insulin resistance.

Patients harbouring *SIM1* RSVs had BMIs of 4.3 and 5.6 SDS, increased longitudinal growth (+0.7 and +1.3 SDS over standardized target height), mild hyperphagia, insulin resistance, and impaired glucose tolerance.

Patients with RSVs in the *BDNF* gene had BMIs ranging from +3.3 to +6.1 SDS. Patients with PPARG RSVs including one sibling (BMI +4.1 to +5.5 SDS) showed insulin resistance and three out of four had dyslipidaemia.

### Replication cohort

The results of the VLF study are shown in Table 1 and Supplemental Table 4. The frequency of RSVs in VLF replicated the results for *BDNF*, *FTO*, *MC3R*, *MC4R* and *SIM1*. No RSVs with the established criteria were detected in *NEGR1* or *PPARG* in the VLF cohort, but likely pathogenic RSVs were identified in *GHSR* and *TMEM18*, absent in controls. The increased rate of RSVs between VLF subjects (32/293, 10.92%) and controls (31/480, 6.46%) in all genes was similar to that of the Spanish EOO dataset (OR = 1.69; *p* = 0.0306). The separate analysis of the overrepresented subset of genes (*BDNF*, *FTO*, *MC3R*, *MC4R*, *NEGR1*, *PPARG* and *SIM1*) yielded a significantly higher proportion of VLF patients with RSVs (16/293) than controls (4/480; OR = 6.55; *p* = 0.0002). This difference was also statistically significant when considering only likely pathogenic variants (6/293 VLF patients versus 0/480 controls; *p* = 0.0029; Supplemental Table 5).

## Discussion

Analysis of an elevated number of individuals is necessary to identify genetic factors with strong effects in heterogeneous diseases with important environmental influence, such as obesity. Pooled DNA-sequencing reduces the number of sequencing reactions, thus reducing costs without decreasing the number of samples. This approach must be optimized to identify all relevant variants and discern between real variants and artefacts. Sequencing coverage above 400 reads in most positions would yield >10 reads of any single RSV diluted in a pool of 20 different samples. Since sequence artefacts are detected in most mapped positions, quality filters were required to discriminate between real variants and false positives. The percentage of reads with the variant, strand bias and Phred score had the greatest discriminatory ability. Full validation rate by Sanger sequencing (23 of 23 variants) proved the high specificity and suitability of the pooling strategy.

We failed to detect one of seven7 previously defined RSVs (sensitivity 86%). This incomplete detection rate might invalidate using pooling strategies for diagnostic purposes, but it should not affect the results of this study. The average coverage of the patient pools was lower than that of the control pools (2396× vs. 3428×). If this difference were to generate bias when detecting RSVs, it would favour RSV detection in controls. A more similar coverage between groups might even increase the difference in burden between patients and controls.

We focused on RSVs in 15 candidate genes that could have a strong effect in a small subgroup of patients according to information available at the onset of this study. This strategy might have missed some variants with higher frequencies that also contribute to obesity. RSVs were identified in 10.36% of patients with half of them (5.0%) most likely being pathogenic, a burden significantly higher in patients than in controls. This burden was attributable to a subset of seven genes (*BDNF*, *FTO*, *MC3R*, *MC4R*, *NEGR1*, *PPARG* and *SIM1*). The replication study corroborated these results indicating that highly penetrant RSVs in these genes contribute to part of the missing heritability of EOO.

When patients are grouped according to the gene mutated, most groups are too small to establish conclusive clinical molecular correlations, but some common features can be discerned. Among the overrepresented genes, *MC4R* was the most frequent (1.51% of patients), similar to reports revealing heterozygous and homozygous mutations in *MC4R* accounting for 1–6% of severe obesity in humans [40, 43, 44]. Patients with RSVs in *MC4R* had hyperphagia with compulsive eating, very severe obesity (mean BMI + 8.9 SDS), overgrowth, accelerated skeletal maturation and metabolic disturbances including insulin resistance. Behavioural disturbances occurred mainly associated with food seeking. It is of note that all patients described here with RSVs at *MC4R* were male. The literature does not support the possibility that in obese subjects this gene is exclusively affected in males. However, there may be a global bias in the parental transmission of RSVs. To determine this possibility, a larger sample size is required, as well as additional investigation. It is also of note that there was a lack of significant differences in gene burden between the two relatively close ethnic groups compared, European Spaniards and Hispanics in the USA.

Like *MC4R*, *MC3R* has a critical role in regulating energy balance [46]. It was recently reported to regulate the upper and lower limits of an individual’s homeostatic set-points and the response to external metabolic challenges [47]. MC3R-knockout mice were shown to have dysregulation of energy expenditure, feeding responses and neuroendocrine responses to metabolic challenges such as fasting, high fat diet intake, pregnancy and the loss of estrogens. For example, these mice lost more weight during fasting than wild-type mice, but gained more weight when put on a high fat diet [47]. The three patients (0.65%) with missense mutations in *MC3R* had a similar degree of obesity, which was milder than those harbouring RSVs in MC4R, and  no overgrowth or advanced skeletal maturation. These patients did not exhibit measurable hyperphagia, similar to what was observed in MC3R-deficient mice [47]. However, as with mice, an inadequate diet could contribute to the excess weight gain. Only one of our patients had hyperinsulinism and hyperuricaemia; this patient also harboured a variant in the other allele that did not fulfil the defined criteria as its frequency is 1/945 in the European population. However, if this second variant is functionally relevant, inheritance would be compatible with a recessive or codominant model as reported for *MC4R*. In the codominant mode of inheritance, both monoallelic and biallelic mutations can cause the disorder and patients with biallelic mutations are more affected than their heterozygous relatives [48].

BDNF is a pro-survival factor in the brain that also participates in appetite regulation. Heterozygous BDNF-knockout mice are obese, hyperphagic and hyperactive [49] and disruption and deletion of *BDNF* have been described in patients with obesity. WAGR syndrome is a contiguous gene deletion syndrome on chromosome 11p13 characterized by Wilms’ tumour, Aniridia, Genitourinary anomalies and Mental retardation, with obesity present in patients with larger deletions encompassing *BDNF* along with the other critical genes (WAGRO syndrome) [10]. Disruption of *BDNF* expression in a patient with a paracentric inversion and deletion of the entire *BDNF* locus in a mother and child were associated with hyperphagia, severe obesity and mildly impaired cognition with or without attention deficits and hyperactivity [50–52]. However, no point mutations in *BDNF* have been reported to date in obese patients. In our series, three patients (0.65%) harboured two different missense RSVs in this gene predicted as pathogenic, with none detected in controls. Two unrelated cases carried the same variant (I231V) and another carried a de novo variant (C141G9). Obese patients with *BDNF* variants presented mild to severe hyperphagia and insulin resistance with dyslipidaemia in two cases. No behavioural or learning issues detected.

Six RSVs in *SIM1* were found in our cohort and two were predicted as likely pathogenic. A third case was the affected brother of case 1782, concordant for the Q206K RSV. The phenotype of all three cases included a BMI-SDS of +4 to +5, no intellectual or behavioural abnormalities, and insulin resistance with impaired glucose tolerance. *Sim1* haploinsufficiency in mice induces hyperphagia, obesity and central nervous system developmental abnormalities [53]. Deletions [54, 55] and translocations [56] affecting *SIM1* cause severe obesity in association with intense orexigenic impulsivity and other features resembling Prader–Willi syndrome. Loss-of-function point mutations in *SIM1* with variable expressivity and incomplete penetrance also produce a “Prader–Willi-like” phenotype [57], but also non-syndromic obesity [58]. Thus the described RSVs could contribute to the postulated increased intra-family risk for non-syndromic obesity [58].

Significant differences in RSV load between patients and controls were detected in *NEGR1* (3 patients, 0 controls), *FTO* (4 patients, 1 control) and *PPARG* (3 patients, 0 controls), with five of the RSVs predicted as likely pathogenic. Although association studies have documented their role in obesity, no highly penetrant RSVs have been described to date in these genes. Our data indicate that some RSVs at *NEGR1*, *FTO* and *PPARG* may behave as highly penetrant alleles causative of EOO, although the number of patients is too small to define distinctive phenotypes. The patient with a likely pathogenic RSV at *NEGR1* had speech and behavioural problems and hyperuricaemia, while the three patients harbouring RSVs in *PPARG* (including one sibling) had insulin resistance and associated dyslipidaemia, in agreement with the role of *PPARG* variants in predisposition to metabolic syndrome [59].

Weaker evidence for definite implication was obtained for *GHSR*, *NTRK2* and *TMEM18*. Likely pathogenic RSVs at *NTRK2* were identified in one of our patients and two from the VLF study, but also in one control. Single patients with RSVs at *GHSR* and *TMEM18*, absent in controls, were found in the VLF cohort. Whether these monoallelic RSVs contribute to the obesity phenotype awaits studies in larger series and functional studies. *De novo NTRK2* mutations, a highly intolerant gene to both loss-of-function (pLI = 1) and missense variants (miss-*z* = 4.35), have been previously associated with severe obesity and developmental delay [17]. Thus, some heterozygous partially functional variants could cause a phenotype of non-syndromic obesity without significant developmental delay.

RSVs in five genes were either absent (*LEP*) or more common in controls than in cases (*ADRB3*, *LEPR*, *PCSK1* and *PCSK2*) in both datasets. Biallelic mutations in *LEP*, *LEPR* and *PCSK1* with a recessive model of inheritance have been described in severely obese patients. In a study of 300 obese subjects, biallelic *LEP* mutations were identified with a frequency of 3% [48], but 3/4th of the patients with biallelic mutations were homozygous, with mutations in identical-by-descent regions in consanguineous families [48]. Thus, the prevalence of biallelic mutations in recessive genes in populations that are not inbred, such as the two cohorts studied here, might be quite low [60]. *POMC* was not included in this study based on the assumed adrenal insufficiency associated to pathogenic variants in this gene. However, *POMC* is now being analysed in this cohort in another study.

It is clear that functional analyses at the cellular, tissue and organism levels are required to define the physiopathogenic mechanisms of all variants reported here. However, we show strong epidemiological and statistical evidence by defining the genes harbouring heterozygous deleterious changes in our large cohort of EOO patients and then validating the results in a replication dataset. Moreover, there is experimental evidence for the abnormal function of some of the reported RSVs [13, 40, 61]. Many genes involved in appetite and metabolic control appear to be sensible to dosage [50, 62, 63], with rescue of haploinsufficiency of *M*c*4r* and *Sim1* recently shown to revert the obese phenotype in mice [64]. Thus, although the molecular mechanisms underlying how these newly described RSVs affect body weight remain to be established, there is precedence for heterozygous affectation of these genes being functionally important. Moreover, the overall prevalence of monoallelic RSVs found in our cohort is similar to that of a recent publication [65] where a diagnosis rate of genetic obesity was reported in 3.9% of patients (48/1230) and 7.3% of the paediatric subgroup (12/164). It should also be emphasized that the interaction of these genetic variants with the environment is of utmost importance with morbid obesity being more likely to occur in these cases when dietary habits are inadequate.

In summary, we documented a higher burden of likely pathogenic heterozygous RSVs in several candidate genes in patients with severe EOO compared to controls. The yield for RSV detection is relatively high, around 5% of patients with EOO. Identification of a potential genetic cause of the phenotype in each case is very important for individualized follow-up and genetic counselling to the family. In addition, definition of the specific defect at the molecular level may be instrumental, as this group of individuals may be relatively refractory to weight loss through diet and exercise and novel therapeutic approaches for genetically defined obesities are becoming available. For instance, highly potent second-generation MC4R agonists lead to weight loss in individuals with MC4R deficiency [40]. Thus, several patients in this study could possibly benefit from this therapy. Our results reinforce the role of the leptin–melanocortin pathway in obesity and bring to light other genes that may carry highly penetrant obesogenic single allele variants, such as *FTO*, *NEGR1* and *PPARG*. The availability of an evidence-based algorithm for diagnostic analysis of early-onset obesity would be of great clinical interest. However, the obese phenotype of the children reported here, as well as in other studies, is non-specific, and an algorithm that could accurately suggest which genes should be studied in each case is difficult even if phenotype–genotype relationships lead to a better definition of the different clinical conditions. Instead, given the currently available and affordable tools for diagnostic genomic analysis, a genotype-first approach using expanded panel of genes with effective copy number analyses should be indicated in these children with early-onset obesity.

## Supplementary information

Supplementary figure legends

Supplemantary information

Supplementary Figure 1

Supplementary Figure 2

Supplementary Table 1

Supplementary Table 2

Supplementary Table 3

Supplementary Table 4

Supplementary Table 5
