# Supplementary material for: Heterozygous rare genetic variants in non-syndromic early-onset obesity
Source: Int J Obes (Lond). 2019 Mar 29;44(4):830–41. doi: 10.1038/s41366-019-0357-5 (PMC7101277; doi:10.1038/s41366-019-0357-5)
Supplement: Supplementary file 1 — Supplementary figure legends [file 41366_2019_357_MOESM1_ESM.docx]

**SUPPLEMENTARY FIGURE LEGENDS**

**Supplementary Figure 1.** Height for age at the age of obesity concern (first visit in our department) in males (A) and females (B) harbouring rare single variants (RSV) in the genes shown by the color key.

**Supplementary Figure 2.** BMI for age at the age of obesity concern (first visit in our department) in males and females harbouring rare single variants (RSV) in the genes shown by the color key. Arrows in red represent that the BMI is over the highest value represented in the charts (BMI-SDS values detailed in table 2).
