# Supplementary material for: Heterozygous rare genetic variants in non-syndromic early-onset obesity
Source: Int J Obes (Lond). 2019 Mar 29;44(4):830–41. doi: 10.1038/s41366-019-0357-5 (PMC7101277; doi:10.1038/s41366-019-0357-5)
Supplement: Supplementary file 2 — Supplemantary information [file 41366_2019_357_MOESM2_ESM.docx]

**SUPPLEMENTARY INFORMATION**

Five Supplementary Tables are included. Table S1 shows the RSVs identified in EOO-Spain patients. Table S2 shows the RSVs in controls. Table S3 includes the constraint metrics of the studied genes. Table S4 shows the RSVs found in Viva la familia patients. Table S5 includes the burden statistics of RSVs.
