# Supplementary material for: Heterozygous rare genetic variants in non-syndromic early-onset obesity
Source: Int J Obes (Lond). 2019 Mar 29;44(4):830–41. doi: 10.1038/s41366-019-0357-5 (PMC7101277; doi:10.1038/s41366-019-0357-5)
Supplement: Supplementary file 3 — Supplementary Figure 1 [file 41366_2019_357_MOESM3_ESM.ppt]

## Slide 1
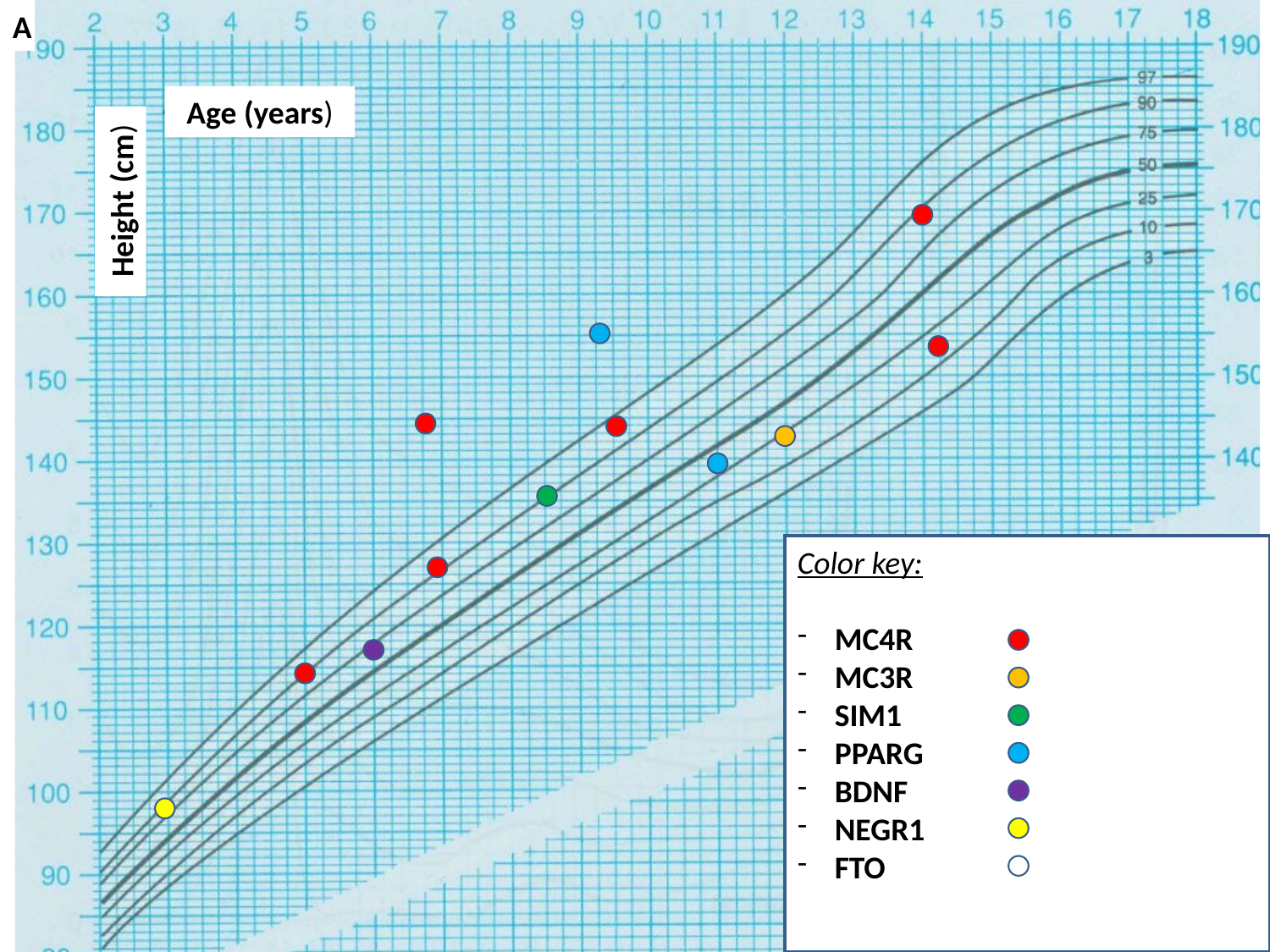

A
Age (years)
Height (cm)
Color key:
 MC4R
 MC3R
 SIM1
 PPARG
 BDNF
 NEGR1
 FTO

## Slide 2
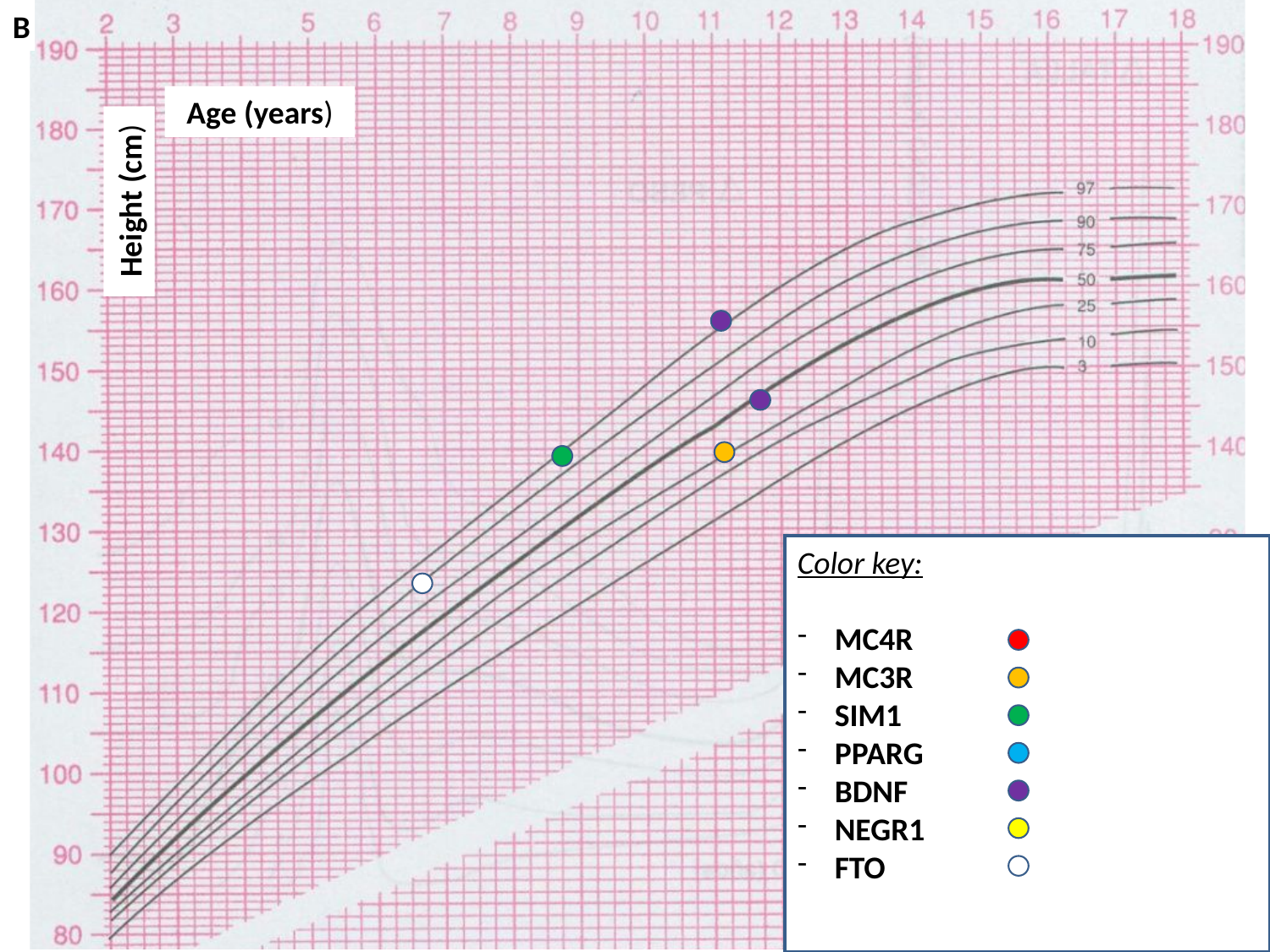

B
Age (years)
Height (cm)
Color key:
 MC4R
 MC3R
 SIM1
 PPARG
 BDNF
 NEGR1
 FTO
