# Supplementary material for: Heterozygous rare genetic variants in non-syndromic early-onset obesity
Source: Int J Obes (Lond). 2019 Mar 29;44(4):830–41. doi: 10.1038/s41366-019-0357-5 (PMC7101277; doi:10.1038/s41366-019-0357-5)
Supplement: Supplementary file 4 — Supplementary Figure 2 [file 41366_2019_357_MOESM4_ESM.ppt]

## Slide 1
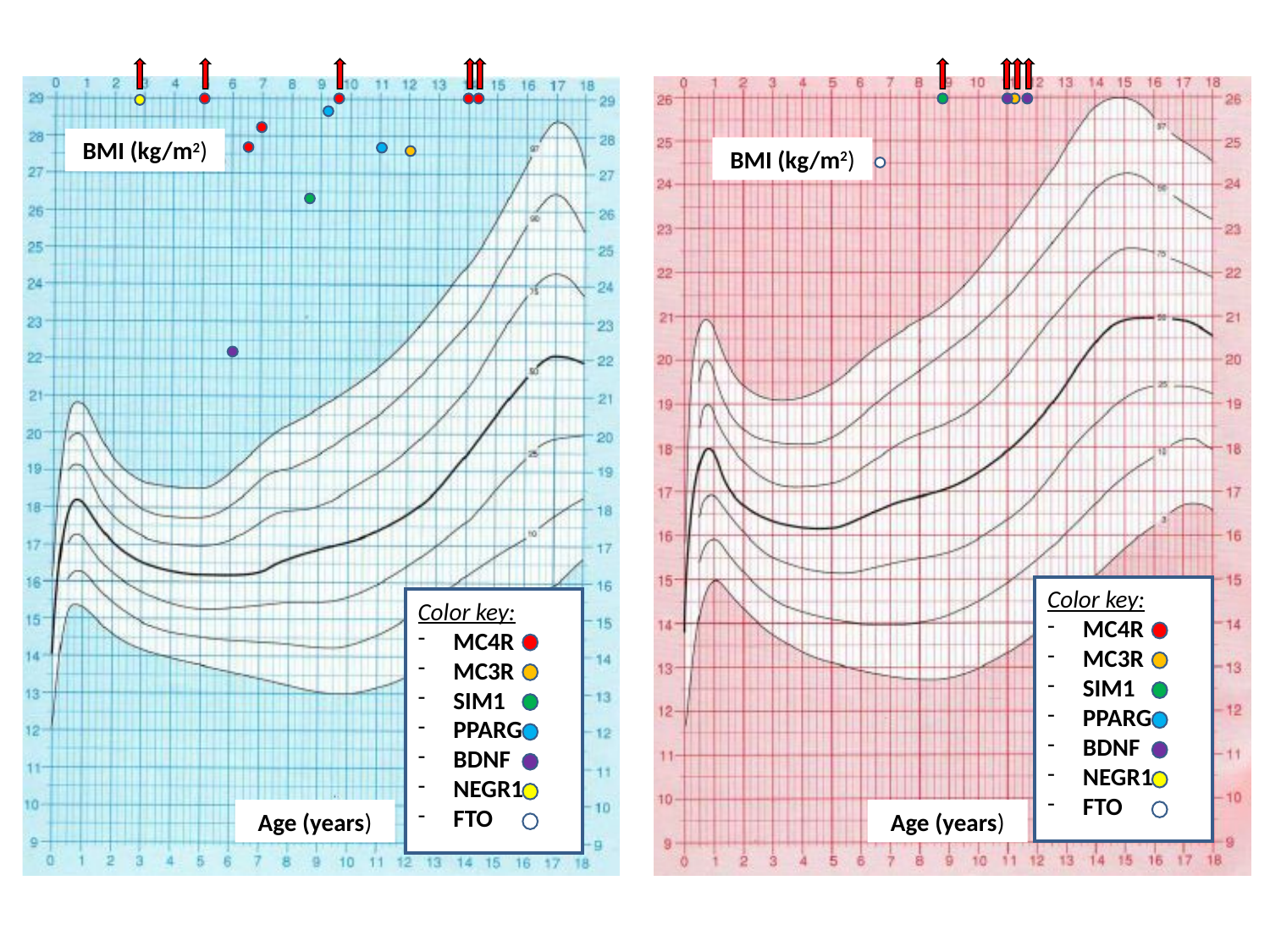

BMI (kg/m2)
BMI (kg/m2)
Color key:
 MC4R
 MC3R
 SIM1
 PPARG
 BDNF
 NEGR1
 FTO
Color key:
 MC4R
 MC3R
 SIM1
 PPARG
 BDNF
 NEGR1
 FTO
Age (years)
Age (years)
